# Improving the support for XML dynamic updates using a hybridization labeling scheme (ORD-GAP)

**DOI:** 10.12688/f1000research.69108.1

**Published:** 2021-09-09

**Authors:** Su-Cheng Haw, Aisyah Amin, Chee-Onn Wong, Samini Subramaniam

**Affiliations:** 1 Faculty of Computing & Informatics, Multimedia University, Cyberjaya, Selangor, 63100, Malaysia; 2 AirAsia Berhad, Lapangan Terbang Antarabangsa Kuala Lumpur (KLIA2), Selangor, 64000, Malaysia

**Keywords:** XML-RDB mapping, mapping scheme, XML databases, dynamic updates, XML labeling scheme.

## Abstract

**Background** **: **As the standard for the exchange of data over the World Wide Web, it is important to ensure that the eXtensible Markup Language (XML) database is capable of supporting not only efficient query processing but also capable of enduring frequent data update operations over the dynamic changes of Web content. Most of the existing XML annotation is based on a labeling scheme to identify each hierarchical position of the XML nodes. This computation is costly as any updates will cause the whole XML tree to be re-labelled. This impact can be observed on large datasets. Therefore, a robust labeling scheme that avoids re-labeling is crucial.

**Method: **Here, we present ORD-GAP (named after Order Gap), a robust and persistent XML labeling scheme that supports dynamic updates. ORD-GAP assigns unique identifiers with gaps in-between XML nodes, which could easily identify the level, Parent-Child (P-C), Ancestor-Descendant (A-D) and sibling relationship. ORD-GAP adopts the OrdPath labeling scheme for any future insertion.

**Results: **We demonstrate that ORD-GAP is robust enough for dynamic updates, and have implemented it in three use cases: (i) left-most, (ii) in-between and (iii) right-most insertion. Experimental evaluations on DBLP dataset demonstrated that ORD-GAP outperformed existing approaches such as ORDPath and ME Labeling concerning database storage size, data loading time and query retrieval. On average, ORD-GAP has the best storing and query retrieval time.

**Conclusion: **The main contributions of this paper are: (i) A robust labeling scheme named ORD-GAP that assigns certain gap between each node to support future insertion, and (ii) An efficient mapping scheme, which built upon ORD-GAP labeling scheme to transform XML into RDB effectively.

## Introduction

Extensible Markup Language (XML) was introduced in the 1990s by the World Wide Web Consortium (W3C) to be the standard for information exchange as it is self-descriptive. Similar to Hypertext Markup Language (HTML), XML is a tag-based syntax, yet, XML can represent data within its context and is readable by machines and humans as it utilizes a natural language. ^1, 2^ Since the emergence of XML, many approaches to map XML into Relational DataBase (RDB) have existed. ^3, 4^

Dynamic Prefix-based Labeling Scheme (DPLS) ^5^ extended the Dewey scheme ^6, 7^ and is based on a two stage approach: (i) constructing the initial DPLS labeling and (ii) handling any updates. Alsubai and North ^8^ proposed a Child Prime Label (CPL) based on the prime number on the XML tree. The trees are traversed and annotated with labels (start, end, level, CPL) based on depth-first traversals. Research by Khanjari and Gaeini ^9^ proposed the FibLSS encoding scheme, which uses binary bit values (0 and 1) to assign node labels. The authors conducted experimental evaluations of their approach against Improved Binary String Labeling (IBSL), ^10^ which indicated that FibLSS is capable of supporting insertion without the need for relabeling.

More recently, Taktek and Thakker ^11^ introduced the Pentagonal Scheme, a dynamic XML labeling scheme. Their algorithms support dynamic updates without redundant labels or relabeling needed. Their evaluations showed that the Pentagonal Scheme can handle several insertions yet showed a better initial labeling time as compared to some existing schemes, especially on large XML datasets. Azzedin *et al.* ^12^ proposed the RLP-Scheme, which enriched Dewey labeling ^6^ with more information. With the RLP-Scheme, an ancestor node can be computed easily, yet the storage space and central processing unit time can be minimised for XML with many identical sub-trees.

In the literature, most of the existing approaches support only static query processing by assuming that the structural information will not have any changes over time. ^13^ This situation is impractical as the data exchanged over the Web is subject to very frequent updates. Due to this reason, we propose a mapping scheme called ORD-GAP that can support updates dynamically. Updates and delete operations are simple as they will not change the existing labeling, thus, the focus of this paper is on the insert operation as insertion will generate new or modify existing labeling.

## Methods

**Figure 1.**
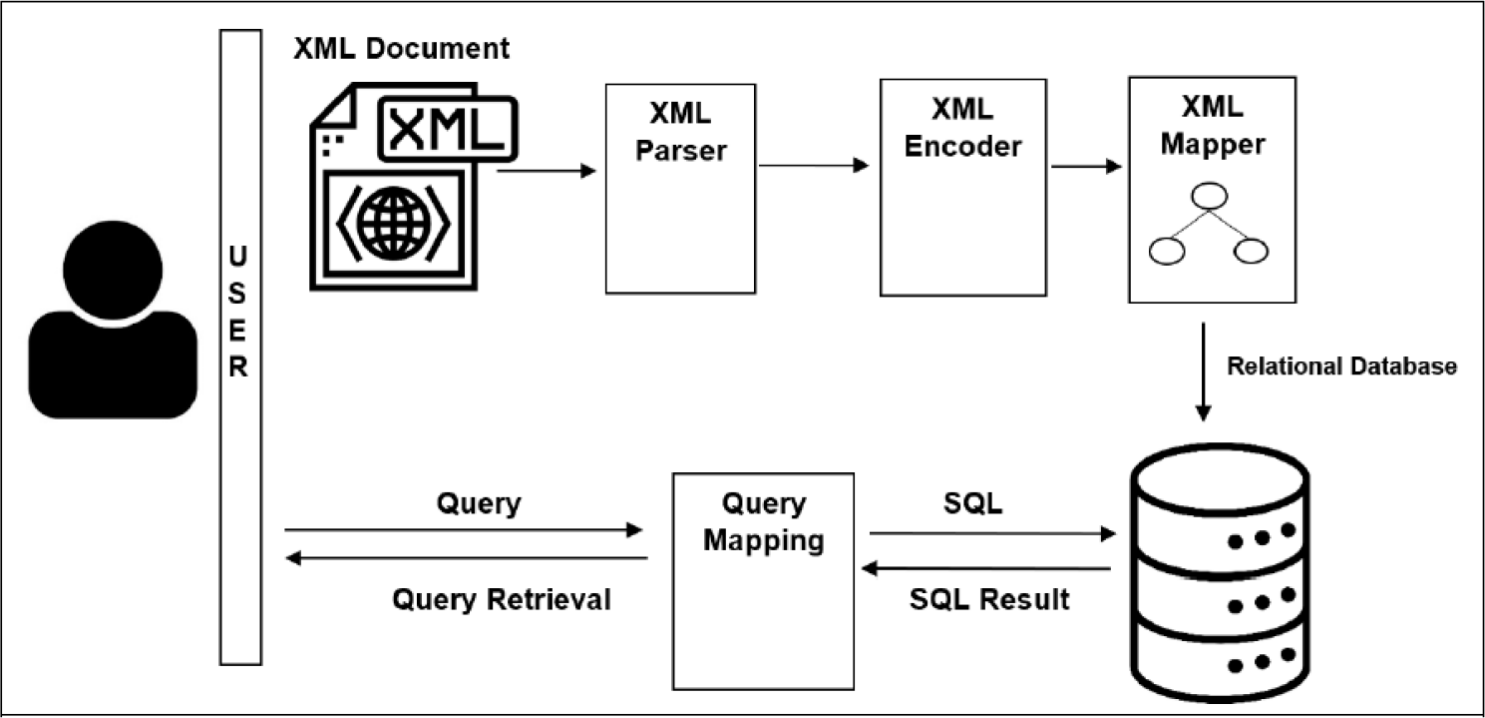
Architecture diagram of the proposed approach.

Figure 1 depicts the architecture diagram of our proposed approach. Our proposed approach consists of the three main components, namely, XML parser, XML Encoder, and XML Mapper. The XML document is the input, while the output will be stored into RDB. The XML parser is responsible for validating XML to ensure it is well-formed before any processing takes place. The XML Encoder annotates the XML tree via a labeling scheme so that the structural relationships among the XML nodes can be identified easily even upon transformation into other underlying storage. Subsequently, the XML Mapper maps or transforms the annotated XML tree into RDB storage. Subsequently, for query retrieval, it will be issued via Structure Query Language (SQL).

### Tree annotation

Tree annotation of the proposed method includes both labeling and mapping schemes that work together to transform the XML tree into RDB storage. This approach adopted the node indexing of range labeling and prefix-based labeling as the initial annotation. Subsequently, we adopted the ORDPath ^14^ labeling scheme for any dynamic update operations. Henceforth, the proposed approach is named as ORD-GAP.

This labeling is in the format of ( *s-e*) *l*. The s denotes the start range while the e denotes the end range. The *l* expresses the level of each node position. These values for *s* and *e* are generated based on the gap *g.* The value *g* is calculated based on the formula: *g*= Σ (max _fan-out_+max _depth_).

**Figure 2.**
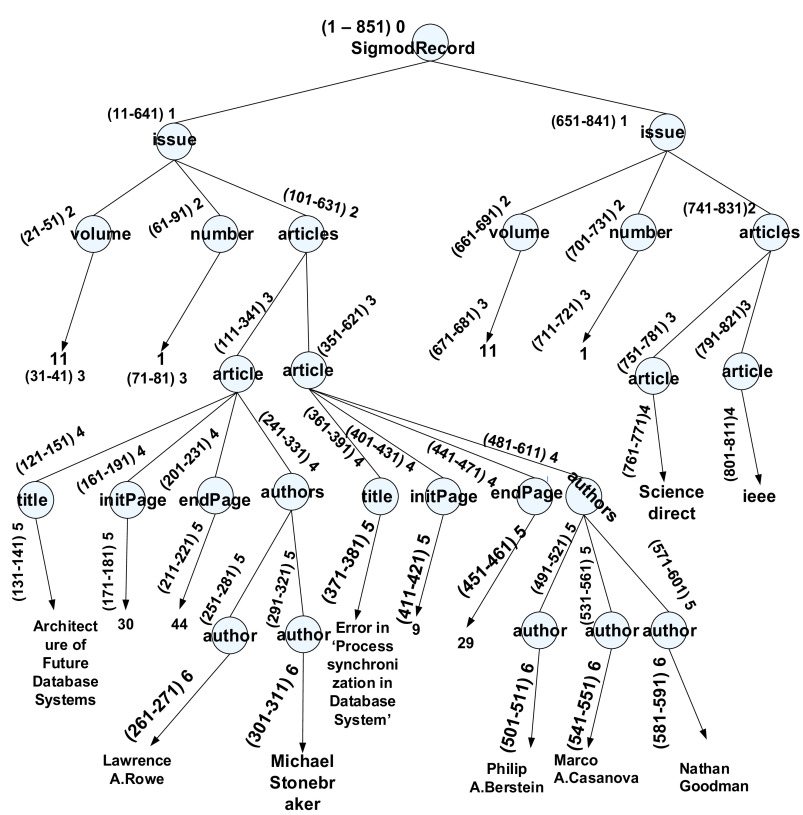
The ORD-GAP labeling scheme.

Figure 2 illustrates the snippet view of the SIGMOD Record dataset ^15^ labelled with the ORD-GAP scheme. This dataset is commonly used for benchmarking purpose. It was chosen as it contains various fan-outs (number of children each node has) and many levels to better demonstrate how our proposed approach works. Firstly, we need to find out the value for *g*, whereby we need to know the max _fan-out_ and max _depth_ From the dataset, we observed that the maximum fan-out and maximum level is 4 and 6 respectively. As such, the gap value calculated by our algorithm (see Figure 3(a)) is 10. The root will always start with *s* as 1. The value of the following node is allocated from the gap value and the previous node’s value. In this case, since the gap is 10 and the value on the previous node’s is 1 (the root node), so, the node “issue” is assigned with 11 and tailed by node “author” with 21 for the *s.* The *e* value on node tree will be assigned once the *s* has reached the leaf node. In this case, if the s label is 31 and is a leaf node, then the *e* label will be assigned with 41 (by adding the *s* value with the gap value, such as 31+11), followed by the node “issue” with 51 as the *e.*

Figure 3 shows the pseudocode for ORD-GAP. Figure 3(a) shows the calculation of *g* which is formulated based on Σ (max _fan-out_ + max _depth_) of the tree while Figure 3(b) shows the algorithm to assign a label. In Function GetGap, parent node and next level of current node is an input used to obtain *g.* The max _fan-out_ is the maximum number of child while max _depth_ is the deepest level of the tree.

**Figure 3(a).**
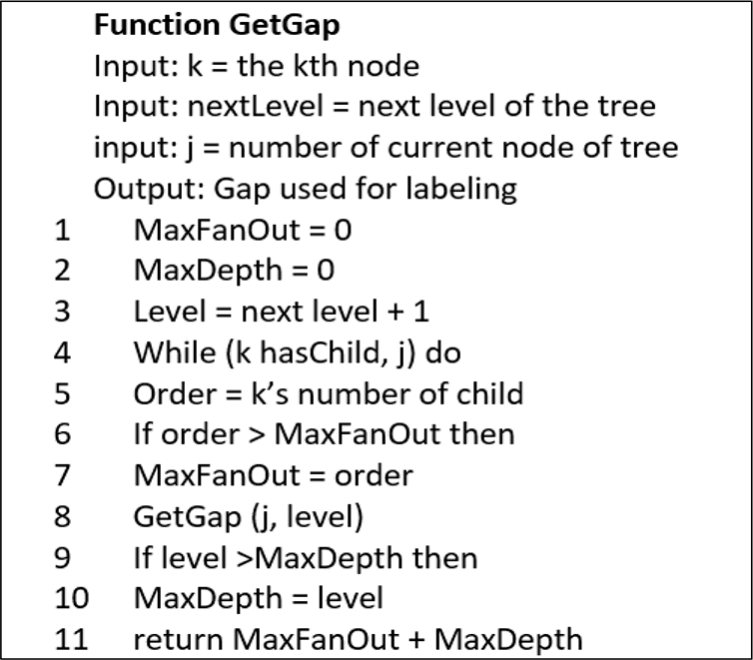
Algorithm for Function GetGap.

**Figure 3(b).**
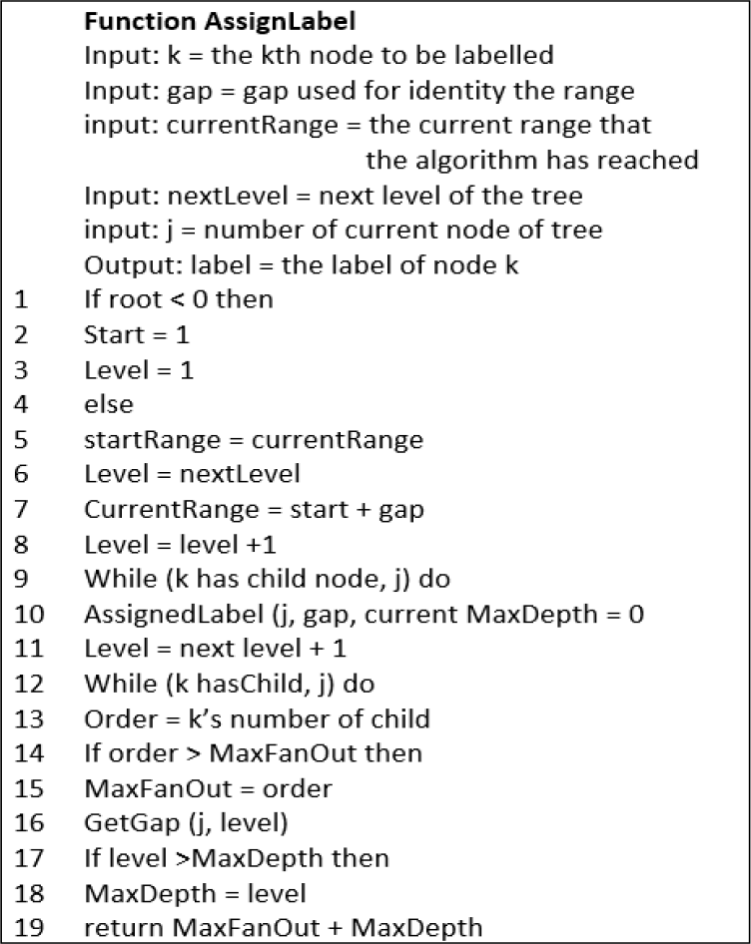
Algorithm for Function AssignLabel

### Structural relationship determination

Mapping schemes of ORG-GAP contain two tables to map the XML data in RDB. The two tables are internal table and text table. The internal table is called iTable, which is used for storing the node that does not contain a text value. A text table is called tTable, and is used to store the leaf nodes. The attributes of both tables consists of Start, End, Level, PStart, Value; Start node keeps the s value of node, End node keeps the e value of node, and Level node keeps the depth of a node from the root. Tables 1 and 2 are the partial view of iTable and tTable based on outcome after the labeling scheme (see Figure 2).

**Table 1.** iTable of Parent Table for initial labeling.

Start	End	Level	Pstart	Value
21	51	2	2	volume
61	91	2	2	number
121	151	4	6	title
161	191	4	6	initPage
201	231	4	6	endPage
251	281	5	10	author
291	321	5	10	author
241	331	4	6	authors
111	341	3	5	article
361	391	4	13	title
401	431	4	13	initPage
441	471	4	13	endPage
491	521	5	17	author
531	561	5	17	author
571	601	5	17	author
481	611	4	13	authors
351	621	3	5	article
101	631	2	2	articles
11	641	1	1	issue
661	691	2	21	volume
701	731	2	21	number
751	781	3	24	article
791	821	3	24	article
741	831	2	21	articles
651	841	1	1	issue
1	851	0	0	SigmodRecord

**Table 2.** tTable of Child Table for initial labeling.

Start	End	Level	Pstart	Value
31	41	3	3	11
71	81	3	4	1
131	141	5	7	Architecture of Future Data Base Systems.
171	181	5	8	30
211	221	5	9	44
371	381	5	14	Errors in 'Process Synchronization in Database Systems'.
411	421	5	15	9
451	461	5	16	29
671	681	3	22	11
711	721	3	23	3
761	771	4	25	science direct
801	811	4	26	ieee

ORD-GAP supports all structural relationships which are level, P-C, A-D and sibling. A-D relationship is determined based on the following conditions:

• if (A(s) < D(s) < A(e)) and (D (level) – A (level) > 1).

**Example:** Let node1 be volume (21-51)2 and node2 be SigmodRecord (1-811)0, (SigmodRecord (1) < volume (21) < SigmodRecord (811) and volume (2) – SigmodRecord (0) > 1). As such, node1 and node2 has A-D relationship.

For P-C relationship, it is determined based on the following conditions:

• if (P(s) < C(s) < P(e)) and (C (level) – P (level) = 1)
• Pstart for C == Start for P (Mapping Scheme)

The level difference should be equal to one since the parent would be only one level higher than the child. Another condition is the PStart value should be equal to P value.

**Example:** Let node1 be article (111-341)3 and node2 be authors (241-331)4, (article (111) < authors (241) < article (341) and authors (4) – article (3)=1). As such, node1 and node2 have P-C relationship.

Lastly for Siblings, if the nodes have the same PStart from the table, they are siblings.

**Example:** Let node1 be endPage (201-231)4 and node2 be authors (241-331)4. From iTable, both have PStart ‘6’. As such, node1 is a sibling of node2.

## Results

The dynamic update of ORD-GAP was adapted from the ORDPath. ^14^ ORDPath encodes the P-C relationship by extending the parent's ORDPath label with a component for the child. However, in ORDPath, the even number is reserved for further node insertions. Generally, this approach is good as all four relationships could be determined easily. However, we observed that the label size grows uncontrollable with the growth of the tree. Henceforth, it may not be scalable for a huge dataset. Yet, we observed that dynamic insertion is not as huge as compared to initial tree labeling. This motivated us to use ORDPath labeling to support the insertion updates, while keeping ORD-GAP as the initial tree labeling.

### Insertion scenario with ORD-GAP

The insertion consists of left-most, right-most and in-between insertion. Each insertion includes an additional node known as medium node which represents the insertion of dynamic update. Thus, this method creates an unlimited insertion on XML tree which avoids node relabeling.

**Figure 4.**
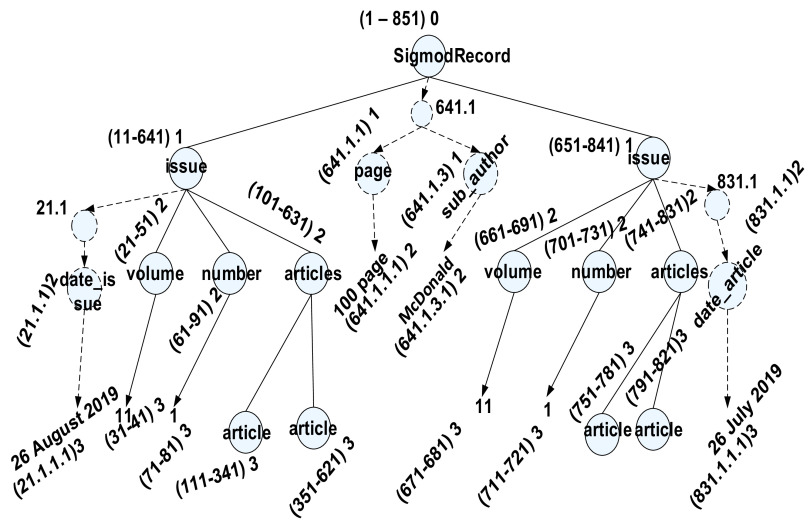
Left-most, in-between and right-most insertion on ORD-GAP.

Figure 4 shows dynamic updates of left-most, in-between, and right-most insertion. The nodes represent the left-most insertion (21.1), in-between insertion (641.1), and right-most insertion (831.1). The insertion contains internal node and leaf node that will be mapped in the iTable (internal table) and tTtable (leaf node) as depicted in Tables 3 and 4, respectively.

**Table 3.** iTable of Parent Table for dynamic updates.

Start	End	Level	Pstart	Value	Pvalue	Type of insertion
21.1	-	1	-	date	issue	Left-most
21.1.1	-	2	-	date_issue	date	Left-most
831.1	-	1	-	date	issue	Right-most
831.1.1	-	2	-	date_article	date	Right-most
641.1	-	0	-	addon	SigmodRecord	In-between
641.1.1	-	1	-	page	addon	In-between
641.1.3	-	1	-	sub_author	addon	In-between

**Table 4.** tTable of Parent Table for dynamic updates.

Start	End	Level	Pstart	Value	Type of insertion
21.1.1.1	-	3	-	26 August 2019	Left-most
831.1.1.1	-	3	-	26 July 2019	Right-most
641.1.1.1	-	2	-	100 page	In-between
641.1.3.1	-	2	-	McDonald	In-between

We have implemented ORD-GAP using Java Development Kit (JDK) 8.0.510.16 on Netbean IDE 8.0.2 compile. Experimental evaluations were conducted to measure the performance of ORD-GAP as compared to ORDPath ^14^ and ME Labeling ^16^ approaches. These two existing approaches were taken for comparison because the technique does not require node re-labeling.

In the first part of the evaluation, the XML document is stored and transformed into RDB storage. The data insertion time and database storage size are recorded for all three approaches. After the storage is completed, we performed query retrieval to measure the performance of ORD-GAP, ORDPath and ME Labeling.

Lastly, our proposed approach ORD-GAP is put into evaluation to test for the dynamic update operations. All the experiments are performed on i7-3770 @3.4 processor with 16GB of RAM running on Windows 7. In the subsequence evaluations, we used the DBLP dataset ^17^ to demonstrate the possibility of supporting larger dataset.

### Data storing evaluation time

In this evaluation, insertion time was recorded four times. We discarded the first reading to omit the buffering effect for consistency of execution time. The results recorded are the average time of the three consecutive times. Table 5 shows the insertion time of ORD-GAP, ORDPath ^14^ and ME labeling. ^16^ ORD-GAP is the fastest followed by ME Labeling and ORDPath.

**Table 5.** XML data insertion on DBLP dataset.

	Insertion time (ms)		
Dataset	ORD-GAP	ORDPath	ME labeling
SigmodRecord	1,926,947	6,111,816	2,491,407

### Storage space evaluation

Database storage consumption was evaluated to determine the storage space using ORD-GAP, ORDPath and ME Labeling approaches. From our experimental observation (see Table 6), we observed that ME Labeling requires higher storage space volume as compared to ORD-GAP and ORDPath due to the larger labeling size required as the depth of the XML tree increases.

**Table 6.** Database sizes of various approaches on DBLP.xml.

Approach	Table	Row	Total row	Database size (KB)	Total database size (MB)
ORD-GAP	iTable	3332130	6337978	401736	749
	tTable	3005848		366088	
ME Labeling	MeParenttable	3332130	6337978	392176	797
	MeChildtable	3005848		424912	
ORDPath	ParentTablereed	3332130	6337978	328264	651
	ChildTablereed	3005848		338448	

As depicted, ORD-GAP reserved a gap between nodes, which delaying the initial node labelling, as ORD-GAP requires some calculation on retrieving the initial nodes. While ORDPath uses dot separated component byte-by-byte, that assigning node label is taken from the parent’s nodes toward the depth of XML tree. Whereas ME Labeling uses multiplication that causes the increases of size labels. The multiplication requires more time on the computation as the size label increase. Thus, both ORDPath and ME Labeling take less time for node labeling.

### Query retrieval evaluation

Table 7 displays the query node in tree representation and XPath notation for each query.

**Table 7.** XPath Notation of DBLP dataset.

Query	Query Node	XPath Notation
PQ1:	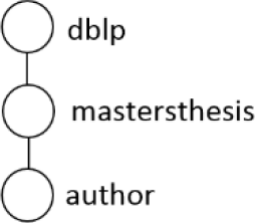	/dblp/mastersthesis/author
PQ2:	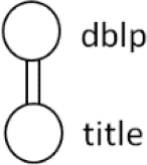	//dblp//title
PQ3:	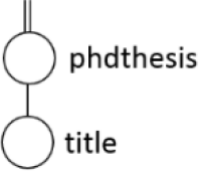	//phdthesis/title
TQ4:	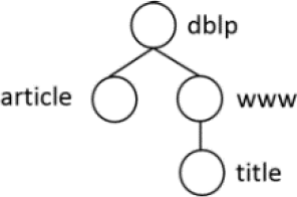	/dblp[/article/www]/title
TQ5:	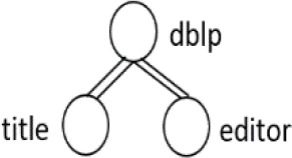	//dblp[//title]//editor
TQ6:	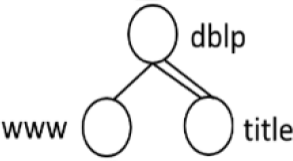	/dblp[/www]//title

**Figure 5.**
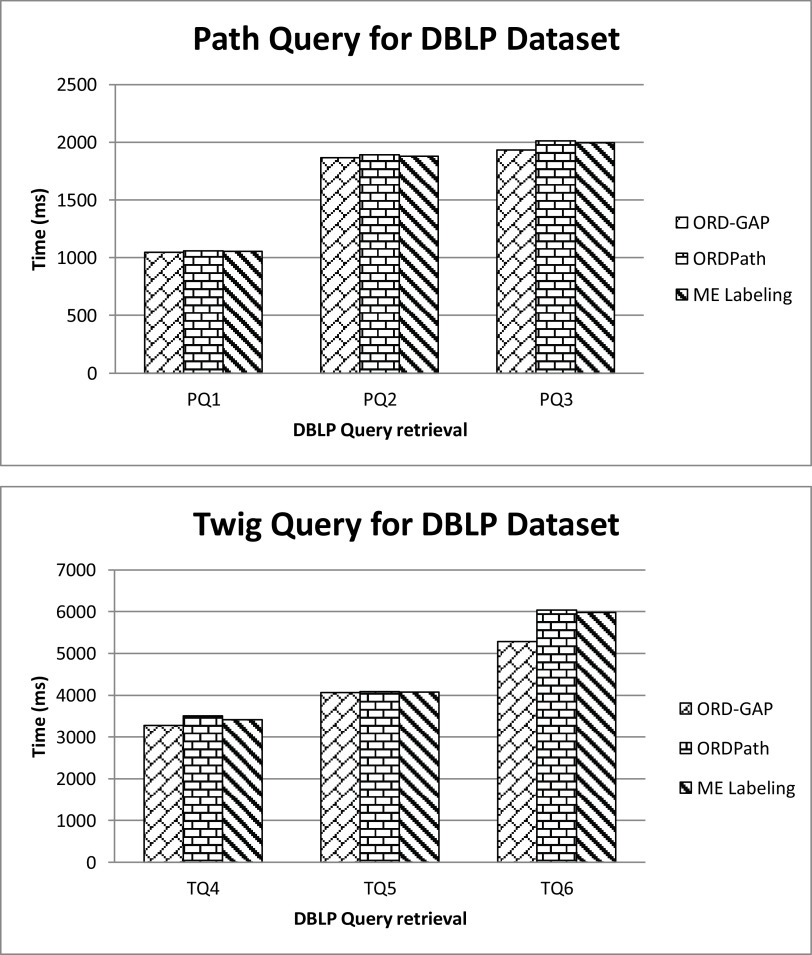
Query retrieval time on DBLP dataset.

Figure 5 shows the query execution performance on various approaches. ORD-GAP is leading, followed by ME labeling and ORDPath. ORDPath require more time as compared to ORD-GAP and ME Labeling due to the number of elements in a node in DBLP. Although DBLP tree contains only three levels, it has multiple siblings in a node. Thus, the data model grows horizontally. ORDPath is prefix-based labeling that traverses using breadth-first search traversal. Likewise, ORDPath did not perform well. As the sibling’s node increases, the size label is increased. Hence, it requires more time to retrieve data in the database.

## Conclusion

In this paper, we propose a labeling scheme named ORD-GAP that enables dynamic insertion by adopting ORDPath techniques. ORDPath generates unrestricted insertion of large XML trees. We carried out evaluations to compare ORD-GAP with ORDPath and ME Labeling. The performance of ORD-GAP was evaluated based on the database size, insertion, query retrieval and dynamic updates. We showed that ORD-GAP has a better performance than ORDPath and ME Labeling. However, we were not able to test ORD-GAP on a dataset size beyond 1.2GB due to hardware limitations such as hardware processor and available RAM.

In our future work, we will look into XML compression and optimization to ensure the further reduce the label size.

## Data availability

### Underlying data

SIGMOD Record dataset available from: http://aiweb.cs.washington.edu/research/projects/xmltk/xmldata/www/repository.html#sigmod-record. ^15^

DBLP dataset available from:

http://aiweb.cs.washington.edu/research/projects/xmltk/xmldata/www/repository.html#dblp. ^17^
