# Supplementary material for: Improving the support for XML dynamic updates using a hybridization labeling scheme (ORD-GAP)
Source: F1000Res. 2021 Sep 9;10:907. [Version 1] doi: 10.12688/f1000research.69108.1 (PMC8772529; doi:10.12688/f1000research.69108.1)
Supplement: Peer review history

# Peer review: Reviewer response for version 1

**DOI:** 10.5256/f1000research.72709.r93981

**Published:** 2021-11-16

**Authors:** Amjad Qtaish

**Affiliations:** 1 College of Computer Science and Engineering, University of Ha’il, Ha’il, Saudi Arabia

This paper proposed a new labeling scheme for solving dynamic XML updates. Three cases of updating (insertions) are used, which are: leftmost, rightmost, and between siblings. I prefer to add another case which is the insertion of the leaf node. In addition, the proposed scheme was evaluated with others in terms of storage size, storage time, and query responses, it outperformed other labeling schemes.

Is the work clearly and accurately presented and does it cite the current literature?

Yes

If applicable, is the statistical analysis and its interpretation appropriate?

Yes

Are all the source data underlying the results available to ensure full reproducibility?

Yes

Is the study design appropriate and is the work technically sound?

Yes

Are the conclusions drawn adequately supported by the results?

Yes

Are sufficient details of methods and analysis provided to allow replication by others?

Yes

Reviewer Expertise:

XML Mapping, Database, Big Data

I confirm that I have read this submission and believe that I have an appropriate level of expertise to confirm that it is of an acceptable scientific standard.

# Peer review: Reviewer response for version 1

**DOI:** 10.5256/f1000research.72709.r93978

**Published:** 2021-09-21

**Authors:** Jiaheng Lu

**Affiliations:** 1 Department of Computer Science, University of Helsinki, Helsinki, Finland

This paper studied the problem of XML update by proposing a new dynamic labeling scheme called ORD-GAP. The methods look effective and the authors perform experiments to verify the update operation and query processing for two datasets: SIGMOD record and DBLP. Experiments show that ORD-GAP has a better performance than ORDPath and ME Labeling. As they mentioned in this article, it would be better to run the experiments on larger data sets, e.g. with Amazon cloud service.

Is the work clearly and accurately presented and does it cite the current literature?

Yes

If applicable, is the statistical analysis and its interpretation appropriate?

Yes

Are all the source data underlying the results available to ensure full reproducibility?

Yes

Is the study design appropriate and is the work technically sound?

Yes

Are the conclusions drawn adequately supported by the results?

Yes

Are sufficient details of methods and analysis provided to allow replication by others?

Yes

Reviewer Expertise:

Databases

I confirm that I have read this submission and believe that I have an appropriate level of expertise to confirm that it is of an acceptable scientific standard.
